# Early Feasibility of Registration of Micro‐PET/CT Scans to Annotated 3D Specimen Models

**DOI:** 10.1002/hed.70363

**Published:** 2026-06-16

**Authors:** Joaquin Austerlitz, Andreja Radevic, Ashtyn McAdoo, Kyrionna Golliday, Cheng Ye, Daniel Fabbri, Adam J. Rosenberg, Marcus Balanky, Anas Alabkaa, Kim A. Ely, Mitra Mehrad, Spencer Roark, Eben Rosenthal, Michael C. Topf

**Affiliations:** ^1^ Department of Otolaryngology – Head and Neck Surgery Vanderbilt University Medical Center Nashville Tennessee USA; ^2^ Department of Chemical and Physical Biology Vanderbilt University Nashville Tennessee USA; ^3^ Vanderbilt University Institute for Imaging Science, Vanderbilt University Medical Center Nashville Tennessee USA; ^4^ Department of Bioinformatics Vanderbilt University Nashville Tennessee USA; ^5^ Department of Radiology and Radiological Sciences Vanderbilt University Medical Center Nashville Tennessee USA; ^6^ Vanderbilt University Medical Center Office of Clinical and Research Safety Nashville Tennessee USA; ^7^ Department of Pathology, Microbiology, and Immunology Vanderbilt University Medical Center Nashville Tennessee USA

## Abstract

**Background:**

Intraoperative ^18^F‐fluorodeoxyglucose (^18^F‐FDG) micro‐positron emission tomography/computed tomography (micro‐PET/CT) is an emerging modality for margin assessment. Prior to clinical use, micro‐PET/CT margin distances must be correlated with gold‐standard histopathology.

**Methods:**

Micro‐PET/CT uptake was co‐registered to 3D specimen models annotated with margin sampling sites using principal component analysis and iterative closest point surface registration. Margin distances were measured on the fused models using Gaussian Mixed Modeling and compared with histopathology.

**Results:**

Fused micro‐PET/CT and 3D specimen models were successfully generated for five patients with head and neck cancer. Among the 16 measured margins, the margin distance on the fused models was within 1 mm of histopathology in 15/16 cases. The Bland–Altman plot demonstrated excellent agreement (±1.25 mm).

**Conclusion:**

We report a novel technique to register micro‐PET/CT data to annotated 3D specimen models. Margin distance measured on the micro‐PET/CT scans correlated closely with histopathology. Intraoperative micro‐PET/CT warrants further investigation in head and neck margin assessment.

## 1 Introduction

Surgical margin status is a critical prognostic factor in head and neck squamous cell carcinoma (HNSCC) [1, 2]. Frozen section analysis (FSA) is utilized intraoperatively to reduce the likelihood of positive surgical margins, which carry an increased risk of recurrence and mortality of 90% [3]. Despite 97% of head and neck surgeons utilizing FSA, this modality is time‐intensive and fails to provide 3‐dimensional (3D) spatial information [4]. Although the main specimen margin status impacts prognosis [5], only 55% of margin specimens are taken via a specimen‐based approach [4]. Tumor‐bed based frozen section margins do not reliably predict the main specimen margin status [6]. This may contribute to the current FSA margin approach failing to improve oncologic outcomes and stagnating positive margin rates in HNSCC [7, 8].

Novel intraoperative approaches such as ^18^fluorine‐fluorodeoxyglucose (^18^F‐FDG) micro‐positron emission tomography/computed tomography (micro‐PET/CT) are being explored as adjuncts to FSA for margin assessment. Prior to meaningful intraoperative use, correlation of uptake on micro‐PET/CT with gold standard histopathology is required. To accurately determine this, margin‐distance measurements made with the micro‐PET/CT must correspond to the exact sites of margin sampling and sectioning. Unfortunately, gross pathologic processing disrupts specimen anatomy, leaving only the written text of the final pathology report and static images to convey anatomy and sites of margin sampling. A modality for the simultaneous visualization of margin sampling sites and micro‐PET/CT data would allow for more precise margin measurement.

We have previously described a method of visualizing the pathologic grossing process with virtual 3D specimen models [9, 10, 11, 12]. In the current study, we describe a method to register micro‐PET/CT Digital Imaging and Communications in Medicine (DICOM) data to the aforementioned 3D specimen model digitally annotated with its pathologic processing. This approach allows for a more precise comparison of micro‐PET/CT margin‐distance measurements with gold standard histopathology margin‐distance measurements. This is used to determine whether histopathology corresponds with ^18^F‐FDG uptake on a micro‐PET/CT, which remains understudied.

## 2 Materials and Methods

This study is performed under Vanderbilt University Medical Center (VUMC) Institutional Review Board approvals (IRB#221597 (3D Scanning) & IRB#242063 (Intraoperative micro‐PET/CT)). This is an ongoing study (NCT06915454) that began in September 2025. Eligible patients are adults (≥ 18 years) with biopsy‐confirmed solid malignancy of the head and neck scheduled to undergo definitive *en bloc* resection with curative intent. Patients with recurrent disease are eligible. Additional eligibility criteria include life expectancy > 12 weeks, Karnofsky performance status ≥ 70% or Eastern Cooperative Oncology Group/Zubrod ≤ 1, and acceptable glucose status (< 200 mg/dL) on the day of surgery prior to radiotracer administration. Exclusion criteria include participation in other research studies involving > 1 millisievert of research‐related radiation exposure within the prior year.

The study protocol is shown in Figure 1.

**FIGURE 1:**
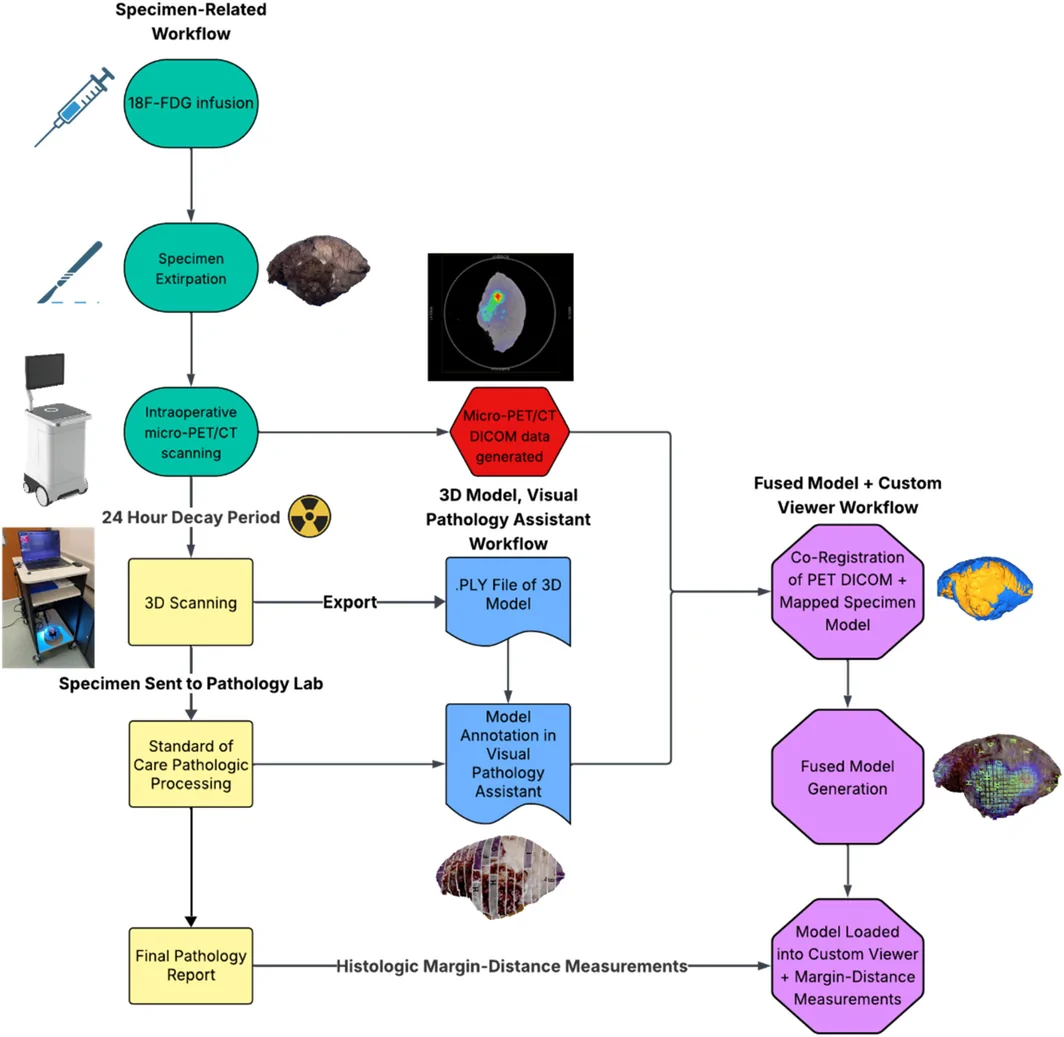
Full fused model generation and measurement workflow. Co‐registration of PET DICOM and the mapped specimen model is performed using centroid alignment, principal component analysis, iterative closest point surface registration, and finally nearest‐neighbor interpolation. [Color figure can be viewed at wileyonlinelibrary.com]

### 2.1 Intraoperative PET/CT

Intraoperative metabolic imaging was performed in the operating room using a high‐resolution mobile micro‐PET/CT scanner (XEOS Aura 10; XEOS Medical, Ghent, Belgium) to assess tumor metabolic activity and support real‐time margin assessment. Image acquisition time for CT images is 2 min with a reconstructed resolution of 0.1 mm. PET acquisition requires 10 min and has an estimated transaxial spatial resolution of 1.87 mm [13]. This represents the full‐width half maximum (FWHM), for example, the distance at which two sources may be independently distinguished on the image.

Participants received a single intraoperative intravenous bolus of ^18^F‐FDG in the operating room 30–90 min prior to specimen extirpation. The injected activity ranged from 44.77 megabecquerel (MBq) to 74.92 MBq; corresponding to a reduced activity relative to standard clinical dosing (e.g., 20% of standard, 0.8 MBq/kg). Following tumor removal, the specimen was oriented and imaged in the operating room by the senior author (M.C.T.) and research team using the micro‐PET/CT device; data acquisition was completed in under 15 min for all cases. For this phase I, open‐label, single‐arm clinical trial, standard of care surgical resection and pathologic processing were not altered by use of the intraoperative micro‐PET/CT. The 3D scanning, alignment, and digital mapping were performed postoperatively for validation. However, much of the alignment and digital mapping process can be performed while the specimen is being processed by the pathology team.

While this manuscript focuses on a novel registration methodology to correlate PET activity with gold standard histopathology, intraoperative margin evaluation was also performed by measuring PET‐derived margin distances directly on the imaging workstation. These intraoperative results will be reported in a separate investigation. After completion of imaging, all resected specimens were placed in formalin and underwent radioactive decay (> 10 half‐lives) followed by the 3D scanning and model annotation protocols. Workflow feasibility and radiation safety compliance were monitored throughout the study and documented. All specimen radioactivity was monitored using a model 9DP Ion Chamber and a model 26‐1 handheld Geiger‐Müller meter (Ludlum Measurements Inc., Sweetwater, Texas, USA) prior to 3D scanning and handling.

### 2.2 3D Scanning

After radioactive decay, resection specimens were prepared for ex vivo surface capture. The specimens' external topography was captured using a commercially available structured‐light 3D scanner and turntable (EinScan SP, Shining 3D, Hangzhou, China) and accompanying software (EXScan, Shining 3D). Specimens were then scanned on each surface until complete external surface coverage was obtained, generating point‐cloud datasets for each surface. Using EXScan, the point clouds were aligned by selecting three corresponding geometric landmarks across datasets, then combined to create a single cohesive reconstruction. The registered dataset was subsequently meshed to produce a watertight, high‐fidelity virtual 3D specimen model and exported as a (.ply) file type. This 3D scanning protocol has been previously published by our team [9, 10, 11]. After completion of 3D scanning, which requires roughly 15 min for scanning and aligning, the specimens were sent for standard of care pathologic processing.

### 2.3 3D Specimen Mapping

The (.ply) files of 3D specimen models were imported into the Visual Pathology Assistant (VPA) custom computer‐aided design (CAD) software, enabling digital annotation of the 3D specimen model. Annotation was performed the day after surgery at the pathology laboratory. Alongside the pathology assistant, a research team member mirrored the standard grossing workflow in the VPA by annotating the digital models in real‐time as the physical specimens underwent inking, sectioning, and processing, thus generating “digital twins” of gross pathologic processing. Inked specimen surfaces were replicated on the virtual model using VPA's brush tool, and cut planes were demarcated using solid white line annotations. Perpendicular sections were visually distinguished using color coding, while shave sections were recorded using a brush‐based marking tool. Each section submitted for histologic analysis was labeled with a letter corresponding to the designated cassette listed in the final pathology report. The full mapping process required 5–10 min added to standard of care processing to ensure accurate model annotation. The resulting 3D specimen maps were reviewed by the pathology assistant and faculty pathologist to verify accuracy. Relevant histopathologic slides were then appended within the VPA software to their corresponding sections with text notes.

### 2.4 Registration of PET/CT DICOM Data and 3D Specimen Map

All data fusion between the intraoperative PET/CT and 3D specimen map was performed using the NumPy and Open3D Python libraries. Prior to the fusion process, the CT DICOM data and the 3D specimen map were first converted into a common file type (.ply). A model of the specimen surface was reconstructed from the intraoperative CT dataset and then registered to the structured‐light‐derived specimen topography. This placed both datasets into a shared coordinate system allowing initial alignment of the datasets using centroid and principal component analysis (PCA). The centroid (geometric center) of each dataset was computed as the average position of all mesh points. Both model centroids were then aligned to achieve approximate co‐localization. PCA was then applied to each dataset to measure the principal axes and estimate directionality of each point cloud. The principal axes were then aligned to achieve directional registration and ensure proper orientation between the CT‐derived surface model and 3D specimen map.

Iterative closest point surface registration was then used to iteratively match corresponding surface points between the two models. This reduced error between paired points and improved alignment precision. PET‐derived metabolic activity data was then transferred to the registered 3D specimen model. PET signal intensity (standardized uptake value, SUV) was mapped onto the aligned 3D mesh using nearest‐neighbor interpolation. For each point or small region on the registered specimen mesh, the nearest corresponding point within the PET dataset was identified, and the SUV value at that PET location was assigned to the mesh point. This resulted in a fused model with spatially mapped metabolic activity. All specimens were 3D scanned following formalin‐fixation, while intraoperative PET/CT imaging was performed prior to formalin fixation. Because the SUV projection is based on weighted‐nearest neighbor interpolation, the mapping of the micro‐PET/CT to the post‐fixation surface effectively compensates for a portion of the size mismatch. Thus, this provides a rough approximation of post‐fixation tissue shrinkage. The fused model was then exported as a (.glb) file type and imported into a custom viewer for interactive margin measurement. The creation of the fused model generated in Case #1 is shown in Figure 2.

**FIGURE 2:**
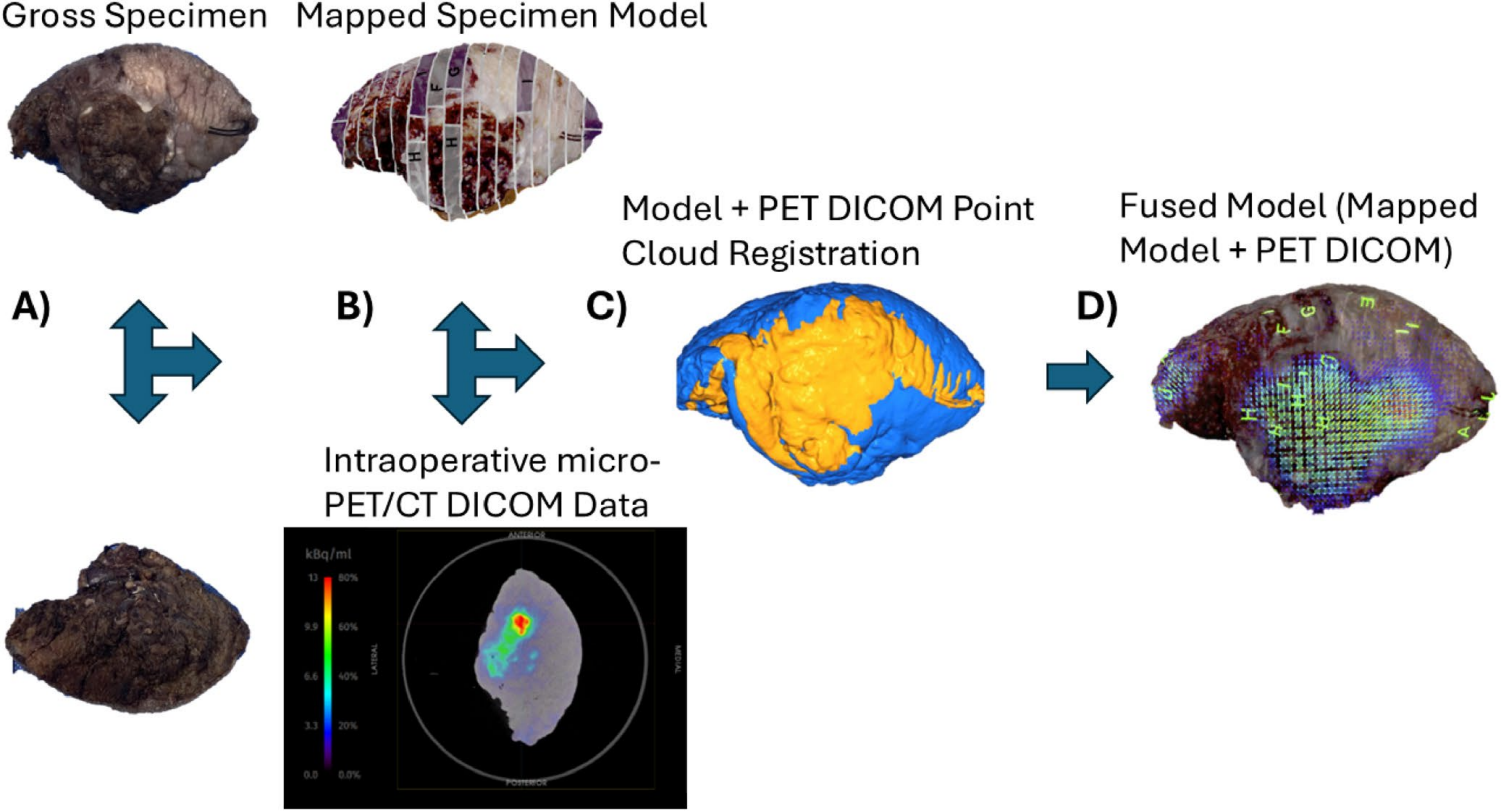
Co‐registration process of micro‐PET/CT DICOM data to right partial glossectomy (Case #1) mapped specimen model to generate fused model (A–D). (A, Top) Mucosal surface view of gross right partial glossectomy specimen following 24‐h formalin fixation and prior to 3D scanning. (Bottom) Deep surface of right partial glossectomy specimen. (B, Top) Mapped right partial glossectomy model (.ply) file. (Bottom) Intraoperative micro‐PET/CT uptake of right partial glossectomy displayed within mobile workstation following extirpation. (C) Aligned point clouds following centroid alignment, principal component analysis, iterative closest point surface registration, and nearest‐neighbor interpolation. (D) Fused model displaying margin sampling sites (letters) and gross anatomy overlaid on micro‐PET/CT uptake. [Color figure can be viewed at wileyonlinelibrary.com]

### 2.5 Margin Selection

The closest margin section and distance is reported in the standard of care written pathology report. Following the release of the final pathology report, the operating surgeon (M.C.T.), attending pathologist, and research team met via teleconferencing. The operating surgeon identified additional clinically relevant margins and histologic margin‐distance measurements were performed by the attending pathologist at that time.

Only a single margin of interest was measured in Cases 1 and 4. In Case 1, during the pathologic grossing process, multiple nearby sections were placed in the same lettered cassettes by the pathology assistant, and the investigators could not determine the exact location on the model that corresponded with the additional measured distances. In Case 4, only a single margin distance was reported on histopathology as the mandible was drilled intraoperatively.

### 2.6 Margin Measurement Within Custom Viewer

A custom 3D model viewer was built in Electron, C++, Rust, and Node.js to support viewing and measuring of the PET cloud within the fused 3D mapped specimen model. SUV data extracted from DICOM data files was registered to a slider bar tool allowing for SUV_max_ thresholding (windowing) of the virtual PET cloud. Intraoperative micro‐PET/CT windowing was standardized at 80% SUV_max_, which was replicated in the custom viewer prior to margin measurement. Accuracy of the PET cloud shape and intensity was then verified visually by comparing to the micro‐PET/CT. Measurements within the tool were performed using ray tracing technology allowing users to click surface points on the mapped model to generate a distance representing the distance from the surface to the first point of ray deflection.

Given the ranging intensities of micro‐PET/CT voxels, Gaussian Mixture Modeling (GMM) was applied to extrapolate SUV values across gaps in voxels while defining SUV‐level boundaries between areas of the PET cloud. This allowed for the determination of distinct groups of voxels and precise SUV and distance values which served as transition points between these groups. This was then clinically correlated using a Kruskal‐Wallis test to determine whether there were significant statistical differences between groups. Groups determined to be significantly different could then be confidently attributed to separate biologic or pathologic processes, that is, background uptake versus tumor uptake. Similar methods have been outlined in lung cancer and served as the basis for this technique [14]. To accommodate GMM segmentation, a radial measurement tool was added to measure multiple points simultaneously. Leveraging the annotations on the mapped model, radial measurements were able to be confined within the sections containing margins of interest. Upon measurement, a GMM plot displaying the boundaries between distinct groups was generated (Figure 3). The SUV and distance values at the points where these transitions occurred were noted on the GMM plot and correlated with gold standard histopathology margin‐distance measurements from the final pathology report.

**FIGURE 3:**
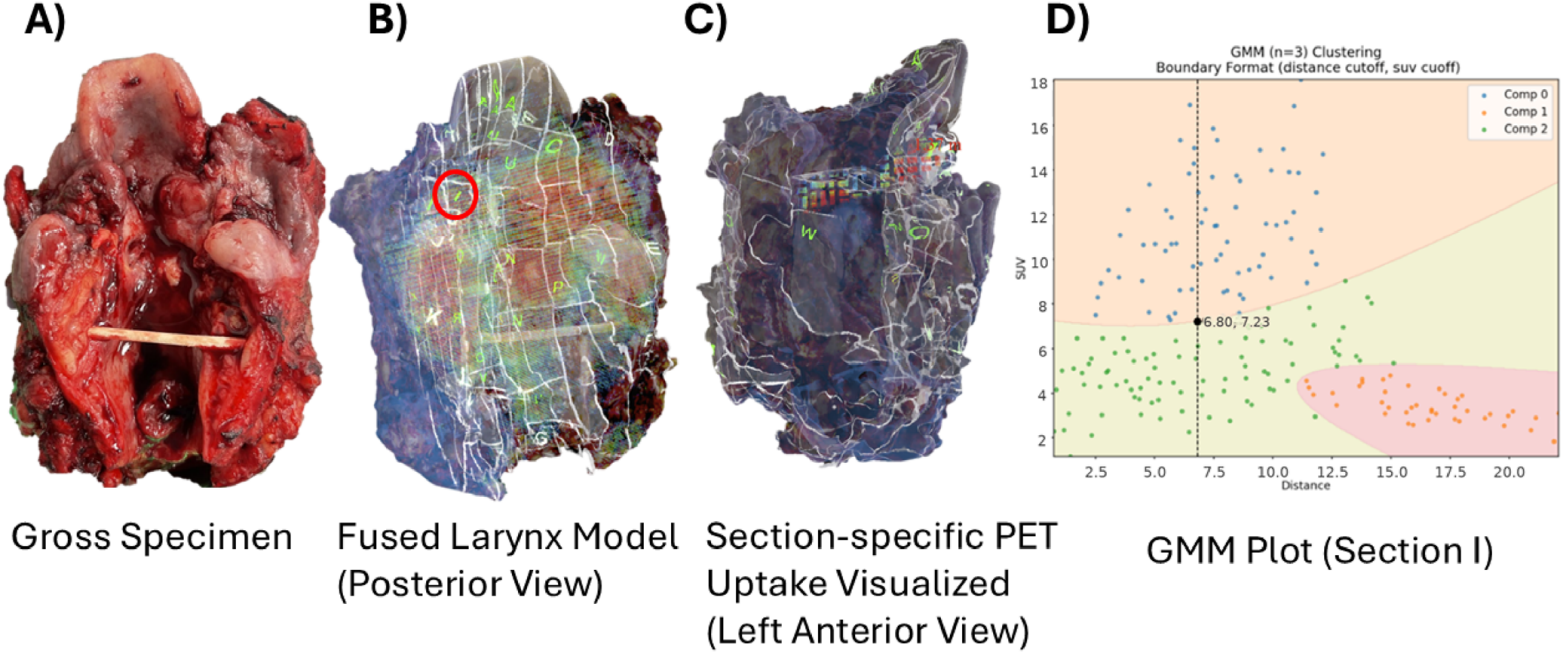
Gaussian mixture model analysis of the left superior aryepiglottic fold margin (section I) from Case #5 (larynx) (A–D). (A) Posterior view of gross total laryngectomy specimen immediately following extirpation (B) Posterior view of the fused larynx model showing margin sampling site section I (red circle) (C) Left anterior view of fused larynx model with “section search” function within custom viewer to restrict visualized micro‐PET/CT uptake to the margin sampling site of interest (section I) (D) GMM plot of micro‐PET/CT voxels within the margin sampling site of interest (section I). [Color figure can be viewed at wileyonlinelibrary.com]

We then performed margin‐distance measurements of the identified margins using the fused model within the custom viewer. The PET windowing parameters were standardized for all measurements 0% of SUV_max_ lower bound, 80% SUV_max_ upper bound. The 80% of SUVmax limit was derived from images acquired and analyzed by Goker et al. [15] and chosen as a reference standard in the subsequent clinical trial [16] to optimize signal intensity across tumor types. All micro‐PET/CT margin measurements were section‐specific by utilizing the annotations of the margins on the fused model. Additionally, all uptake within each margin of interest was included in GMM and Kruskal‐Wallis analysis when determining margin distances within the custom viewer to assess discrimination between true positive and false positive uptake. Therefore, micro‐PET/CT margin measurements correspond with pathologic sections in which the gold standard histopathology is known. Fused model‐derived measurements were compared to gold standard histopathology margin‐distance measurements. Histopathology margin distances at their corresponding sections were measured per standard of care.

### 2.7 Statistical Analysis

The results of GMM analysis of the margin distances using the fused models were compared to gold standard histopathology margin‐distance measurements. All data analysis was performed in R (Posit PBC inc., Boston, Massachusetts, USA). Differences were visually examined for normality (quantile‐quantile plot). Bland–Altman analysis was then performed to estimate mean bias and 95% limits of agreements (95% LoA). A Bland–Altman plot was generated to evaluate agreement between both methods. A linear regression analysis was then conducted to assess the presence of proportional bias. The intraclass correlation coefficient (ICC) for absolute agreement was additionally used to summarize concordance between methods. As multiple measurements were obtained for each case, uncertainty estimates were generated using case‐level bootstrapping.

## 3 Results

Five patients with HNSCC, 3 male and 2 female, underwent intraoperative micro‐PET/CT with fused model generation. The mean age of participants was 53.8 years old. Primary subsites included 2 oral tongue, 1 retromolar trigone, 1 maxillary alveolar ridge, and 1 larynx. These patients underwent partial glossectomy (2), segmental mandibulectomy (1), maxillectomy (1), and total laryngectomy (1), respectively. Perineural invasion was identified in Case 1 while no lymphovascular invasion was identified in any of the cases. Two patients (cases 2 and 4) were previously treated for HNSCC and underwent salvage surgery. One patient received 2 cycles of neoadjuvant pembrolizumab with minimal pathologic response to treatment. Complete demographic and oncologic data are reported in Table 1.

**TABLE 1:** Patient demographic and oncologic information for all cases.

Characteristic	Case 1	Case 2	Case 3	Case 4	Case 5
Sex	Male	Female	Female	Male	Male
Age (Y)	37	60	71	60	41
Tumor subsite	Oral tongue	Retromolar trigone	Oral tongue	Maxillary alveolar ridge	Larynx
Histology	SCC	SCC	SCC	SCC	SCC
Prior oncologic treatment	No	Left oral composite resection	No	Definitive chemoradiotherapy	No
Pathologic T stage	pT3	pT1	pT2	pT2	ypT4a
Greatest tumor dimension (cm)	3.1	0.3	2.7	2.7	4.9
Specimen SUV_max_	29	3.6	5.1	15	39
Closest margin distance (mm)	7	5	3	5	0

Abbreviation: SCC, squamous cell carcinoma.

### 3.1 Intraoperative PET/CT

Patients received an average dose of 55.5 MBq (SD 9.25, range 42.55–62.9 MBq) from infusion of the ^18^F‐FDG tracer. Average time from infusion to specimen extirpation was 88.0 min (SD 79.2, range 28–225 min). Mean SUV_max_ of the 5 specimens was 18.3 (SD 15.4, range 3.6–39).

### 3.2 Fused Model Measurements

Sixteen margin‐distance measurements were made across 5 fused models. Case 1 and 4 had a single margin distance measured while Cases 2, 3, and 5 had 2, 6, and 6 margin distances measured, respectively. Measurements were all performed using standardized windowing lower bound of 0% of SUV_max_ and upper bound of 80% of SUV_max_. Median measured margin distance was 5.8 mm (IQR 4.5–7.5 mm, range 0.75–10.3 mm). All margin‐distance measurements are shown in Table 2. The 3D models for each case are shown in Figures 4, 5, 6, 7, 8. Representative intraoperative micro‐PET/CT images of measured margins and their histology are shown in Figures 9 and S1–S4.

**TABLE 2:** Measured margin distances for all cases on gold standard histopathology and fused models with signed difference (mm).

Case	Anatomic margin	Histopathology (mm)	Fused model (mm)	Difference (Fused−Histo) (mm)
Glossectomy (1)	Deep	7.00	6.50	−0.50
Mandible	Medial	5.00	5.70	0.70
	Lateral	6.00	7.60	1.60
Glossectomy (2)	Lateral	5.00	5.30	0.30
	Posterior	3.00	3.20	0.20
	Anterior	6.00	5.90	−0.10
	Deep	5.00	4.50	−0.50
	Medial	10.00	10.30	0.30
	Medial	10.00	9.70	−0.30
Maxilla	Lateral	5.00	5.50	0.50
Larynx	Left hypopharynx	0.00	0.75	0.75
	Right aryepiglottic fold	3.50	4.00	0.50
	Left aryepiglottic fold	7.00	6.90	−0.10
	Right anterior soft tissue	8.00	7.40	−0.60
	Superoanterior soft tissue	9.00	8.30	−0.70
	Superoanterior soft tissue	3.50	4.40	0.90

**FIGURE 4:**
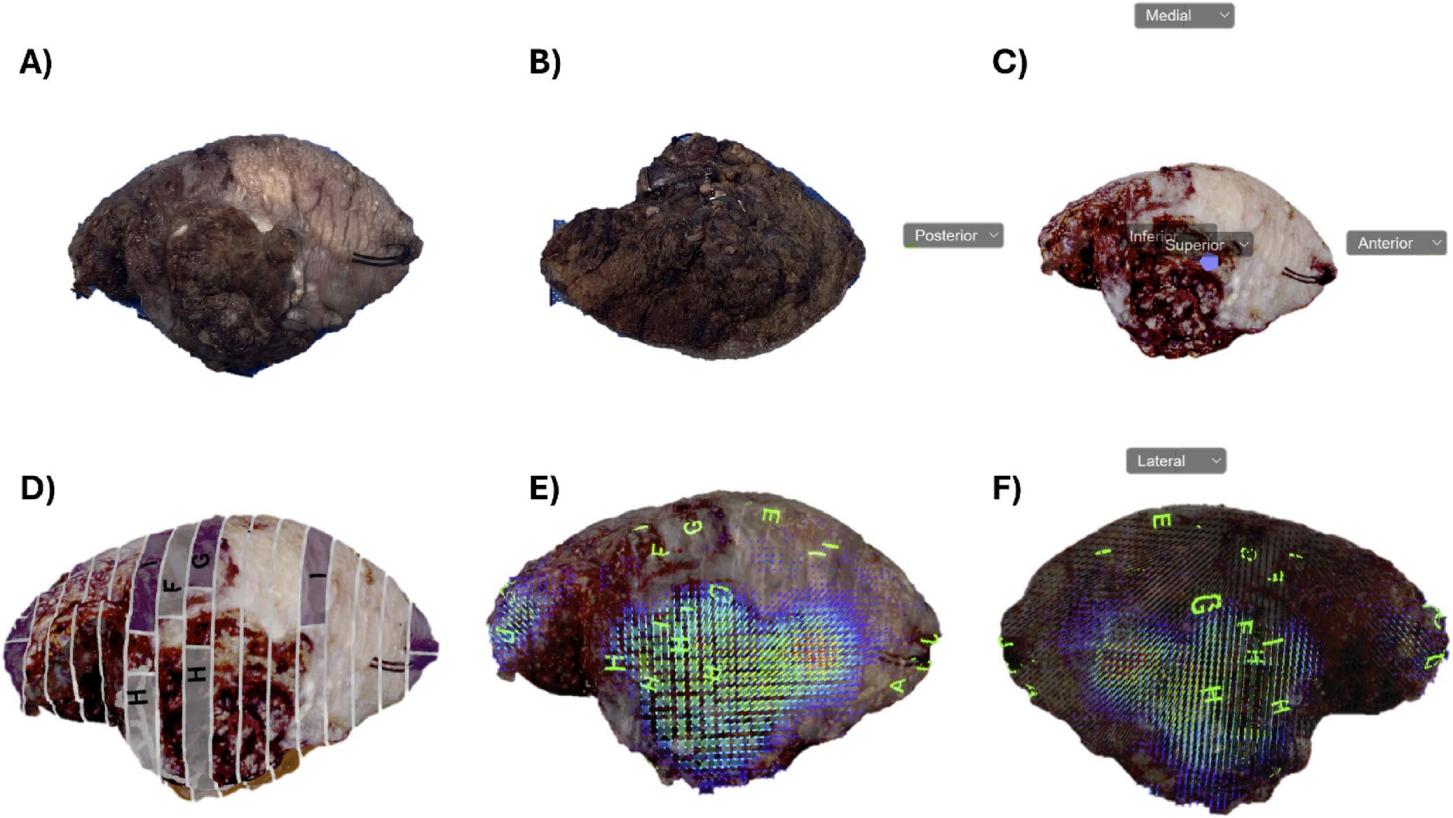
Gross specimen, 3D specimen models (unmapped and mapped), and fused models of Case #1 (right partial glossectomy) (A–F). (A) Mucosal surface view of gross right partial glossectomy specimen following 24‐h formalin fixation. (B) Deep surface view of gross specimen. (C) Unmapped 3D specimen model of right partial glossectomy specimen with orientation axes displayed within the Visual Pathology Assistant software. (D) Mapped 3D specimen model. White and purple shaded sections denote tissue was submitted for histologic analysis while letters correspond to tissue cassettes. (E) Fused model (mucosal surface) with overlaid micro‐PET/CT uptake and highlighted letter markers. (F) Fused model (deep surface). [Color figure can be viewed at wileyonlinelibrary.com]

**FIGURE 5:**
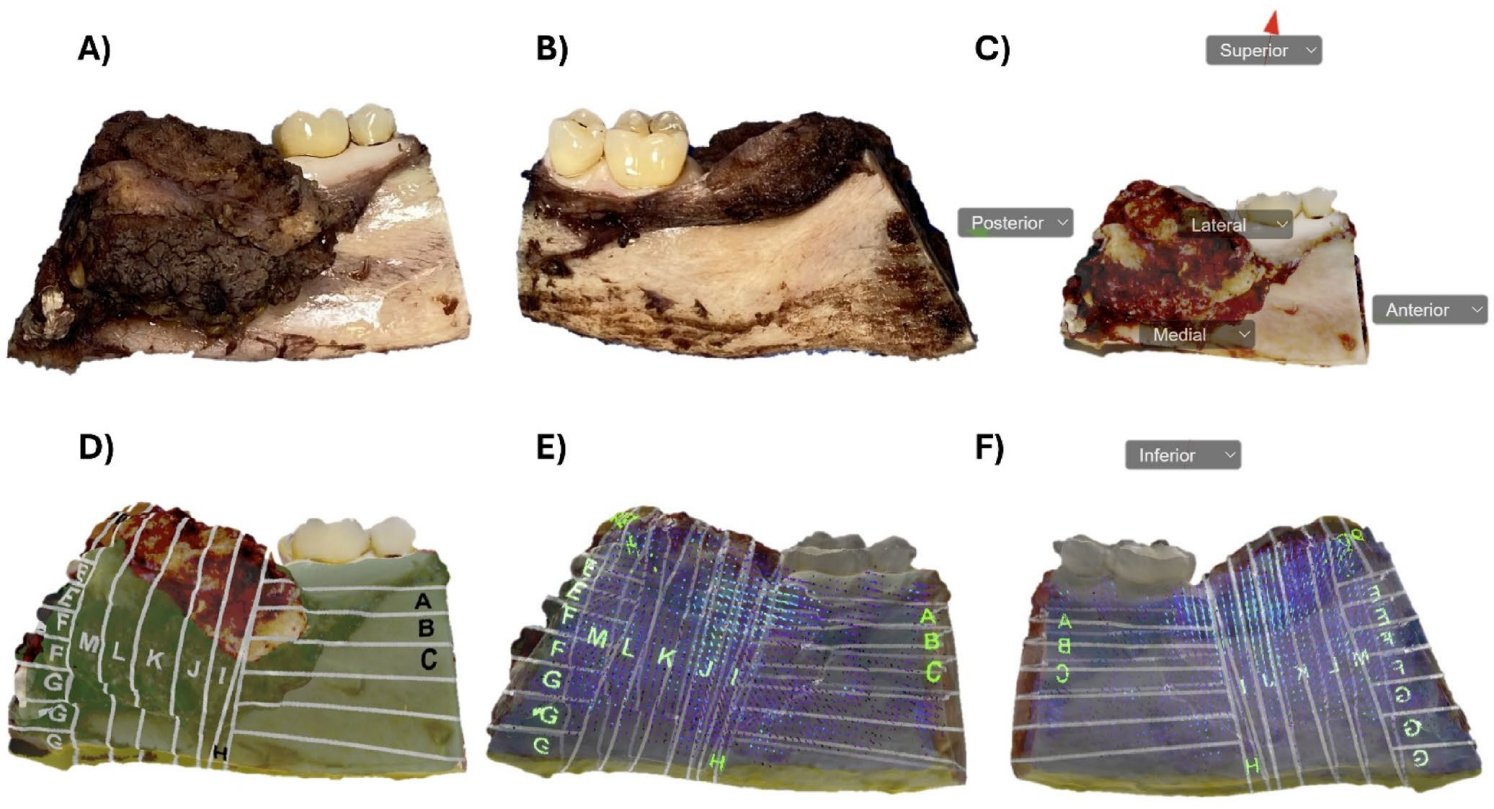
Gross specimen, 3D specimen models (unmapped and mapped), and fused models of Case #2 (left mandibulectomy) (A–F). (A) Medial surface view of gross left mandibulectomy specimen following 24‐h formalin fixation. (B) Lateral surface view of gross specimen. (C) Unmapped 3D specimen model of left mandibulectomy specimen with orientation axes displayed within the Visual Pathology Assistant software. (D) Mapped 3D specimen model. The specimen was submitted entirely for histopathology, thus shading was not used to preserve visualization of anatomy. Letters correspond to tissue cassettes. (E) Fused model (medial surface) with overlaid micro‐PET/CT uptake and highlighted letter markers. (F) Fused model (deep surface). [Color figure can be viewed at wileyonlinelibrary.com]

**FIGURE 6:**
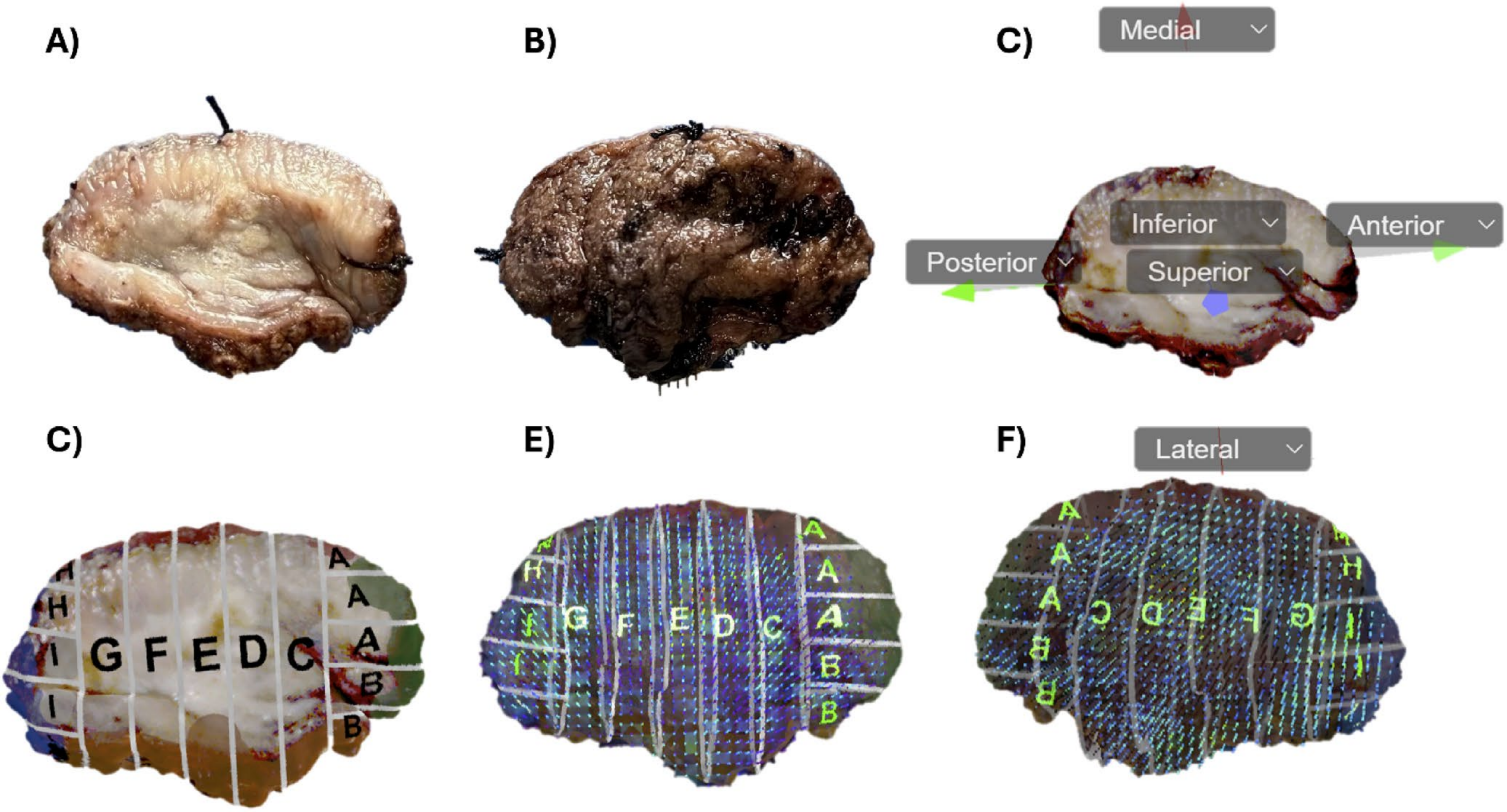
Gross specimen, 3D specimen models (unmapped and mapped), and fused models of Case #3 (right partial glossectomy) (A–D). (A) Mucosal surface view of gross right partial glossectomy specimen following 24‐h formalin fixation. (B) Deep surface view of gross specimen. (C) Unmapped 3D specimen model of right partial glossectomy specimen with orientation axes displayed within the Visual Pathology Assistant software. (D) Mapped 3D specimen model. The specimen was submitted entirely for histopathology, thus shading was not used to preserve visualization of anatomy. Letters correspond to tissue cassettes. (E) Fused model (mucosal surface) with overlaid micro‐PET/CT uptake and highlighted letter markers. (F) Fused model (deep surface). [Color figure can be viewed at wileyonlinelibrary.com]

**FIGURE 7:**
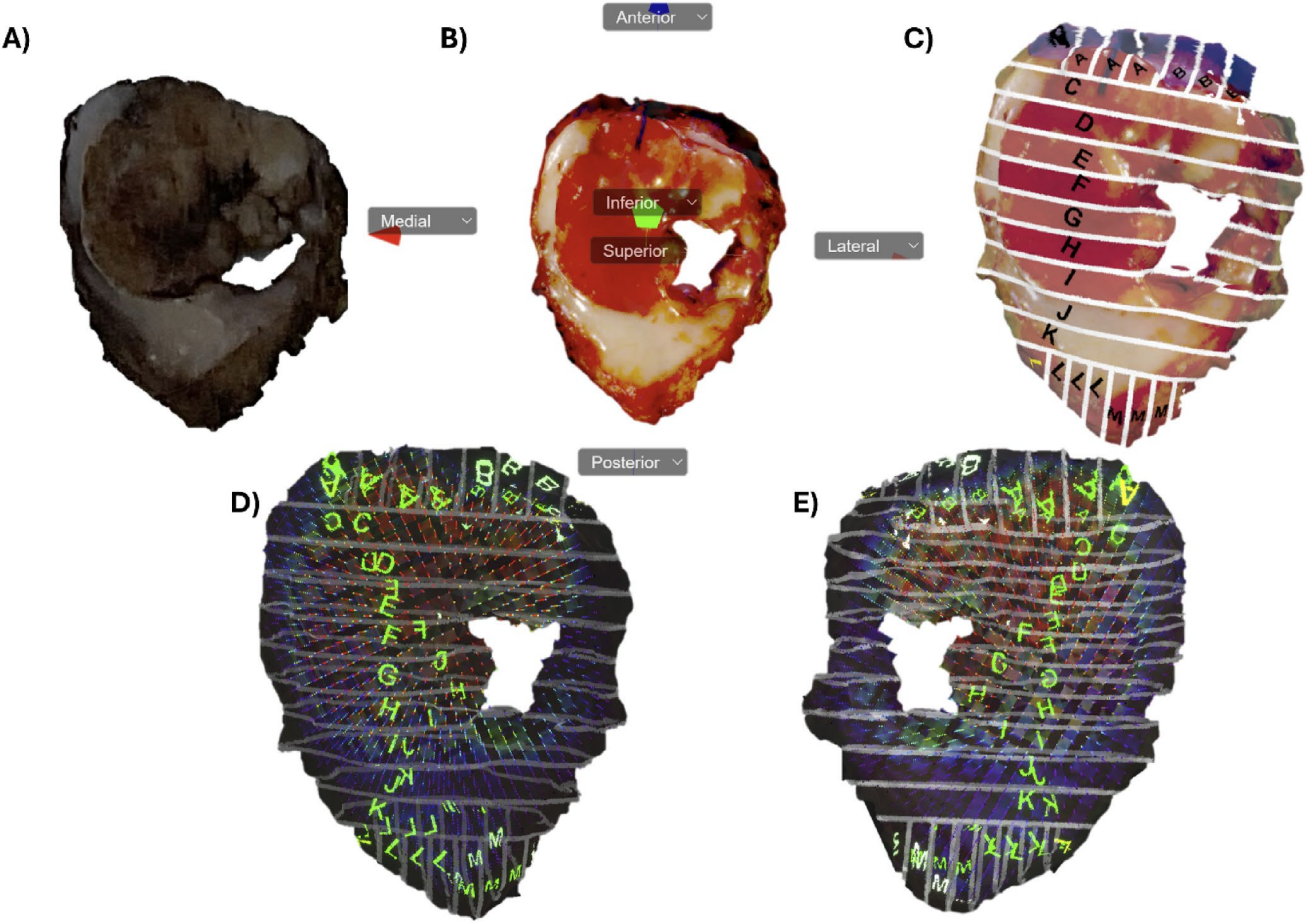
Gross specimen, 3D specimen models (unmapped and mapped) and fused models of Case #4 (left maxillectomy) (A–E). (A) Mucosal surface view of gross left maxillectomy specimen following 24‐h formalin fixation. (B) Unmapped 3D specimen model of left maxillectomy specimen with orientation axes displayed within the Visual Pathology Assistant software. (C) Mapped 3D specimen model. White shaded sections were submitted for histopathology while letters correspond to tissue cassettes. (D) Fused model (mucosal surface) with overlaid micro‐PET/CT uptake and highlighted letter markers. (E) Fused model (deep surface). [Color figure can be viewed at wileyonlinelibrary.com]

**FIGURE 8:**
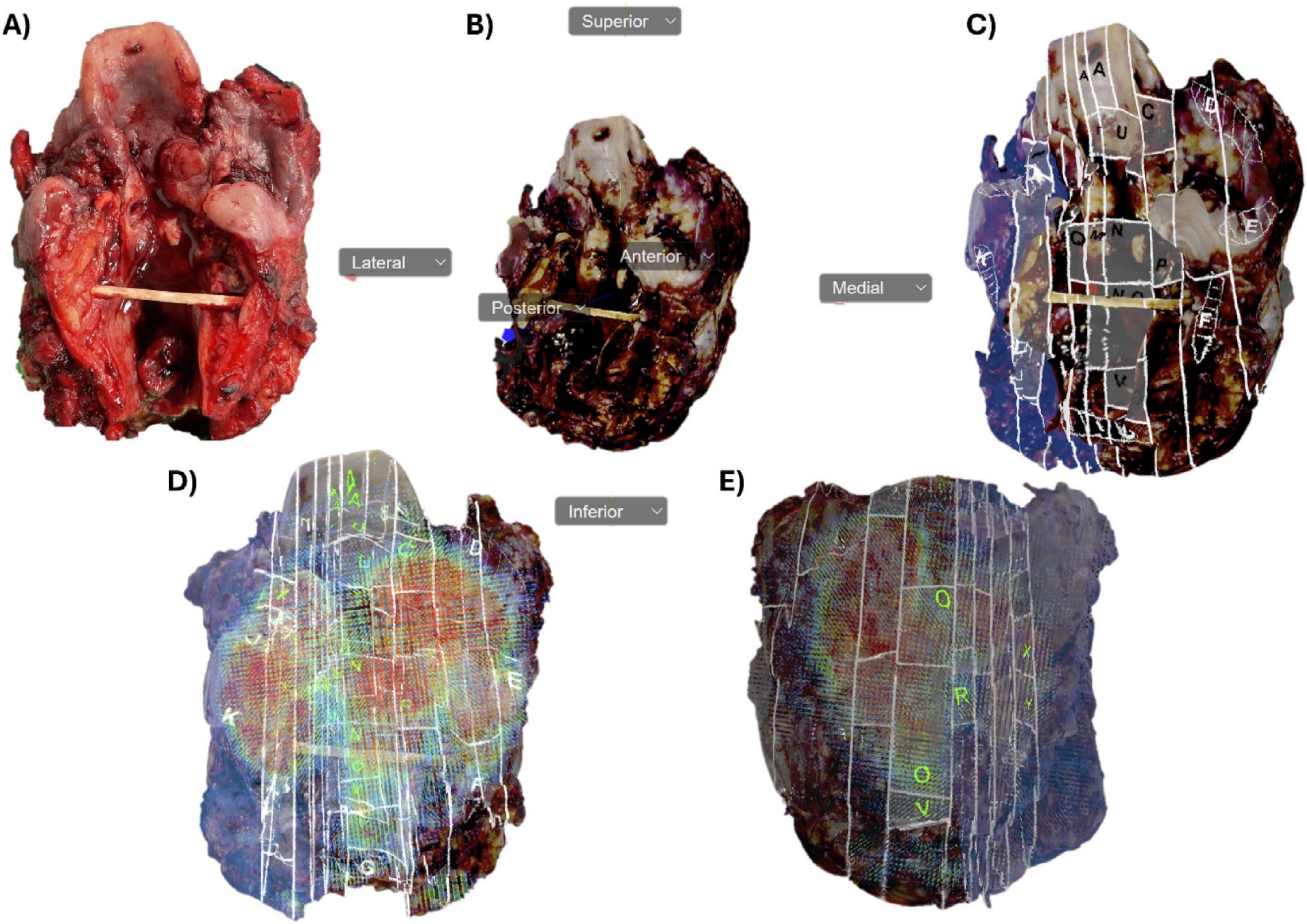
Gross specimen, 3D specimen models (unmapped and mapped), and fused models of Case #5 (total laryngectomy) (A–E). (A) Posterior view of gross total laryngectomy specimen immediately following intraoperative micro‐PET/CT imaging. (B) Unmapped 3D specimen model of total laryngectomy specimen with orientation axes displayed within the Visual Pathology Assistant software. (C) Mapped 3D specimen model. White and purple shaded sections were submitted for histopathology while letters correspond to tissue cassettes. (D) Fused model (posterior/mucosal surface) with overlaid micro‐PET/CT uptake and highlighted letter markers. (E) Fused model (anterior/soft tissue surface). [Color figure can be viewed at wileyonlinelibrary.com]

**FIGURE 9:**
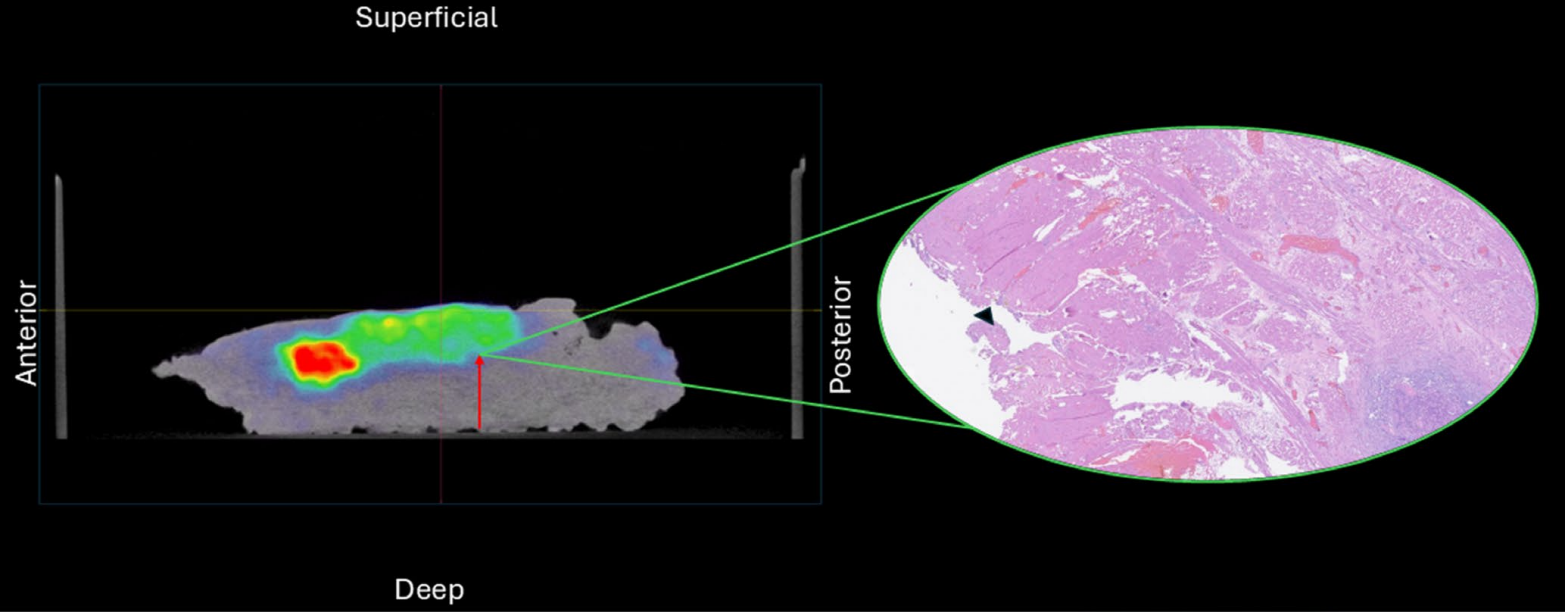
Sagittal image on intraoperative micro‐PET/CT and histopathology of Case #1 deep margin (Left–Right). (Left) Sagittal slice of a right partial glossectomy specimen (Case #1) from intraoperative micro‐PET/CT demonstrating the deep margin (red arrow). (Right) Histopathology of the deep margin (black arrow) from final pathology report. [Color figure can be viewed at wileyonlinelibrary.com]

Sixteen paired margin‐distance measurements (mm) were compared between fused models in the custom viewer and gold standard histopathology. Of the 16 paired margin measurements, 62.5% (10/16) differed by ±0.5 mm while 93.8% (15/16) were within ±1.0 mm. The only micro‐PET/CT margin distance that differed by more than 1 mm from the ground truth margin distance was the lateral margin in Case 2 (mandibulectomy, 1.6 mm). There was a slightly greater underestimation by the fused‐model method at larger margin distances (slope −0.10 mm per 1 mm, *p* = 0.11) on proportional‐bias assessment.

The Bland–Altman plot demonstrated excellent agreement between methods with an average difference (bias) in paired measurements of +0.18 mm and 0.97 absolute‐agreement of measurements by ICC. At an individual level, 95% LoA indicated that fused model‐derived measurements deviated by ±1.25 mm of gold standard histopathology (−1.06 to +1.43 mm) (Figure 10).

**FIGURE 10:**
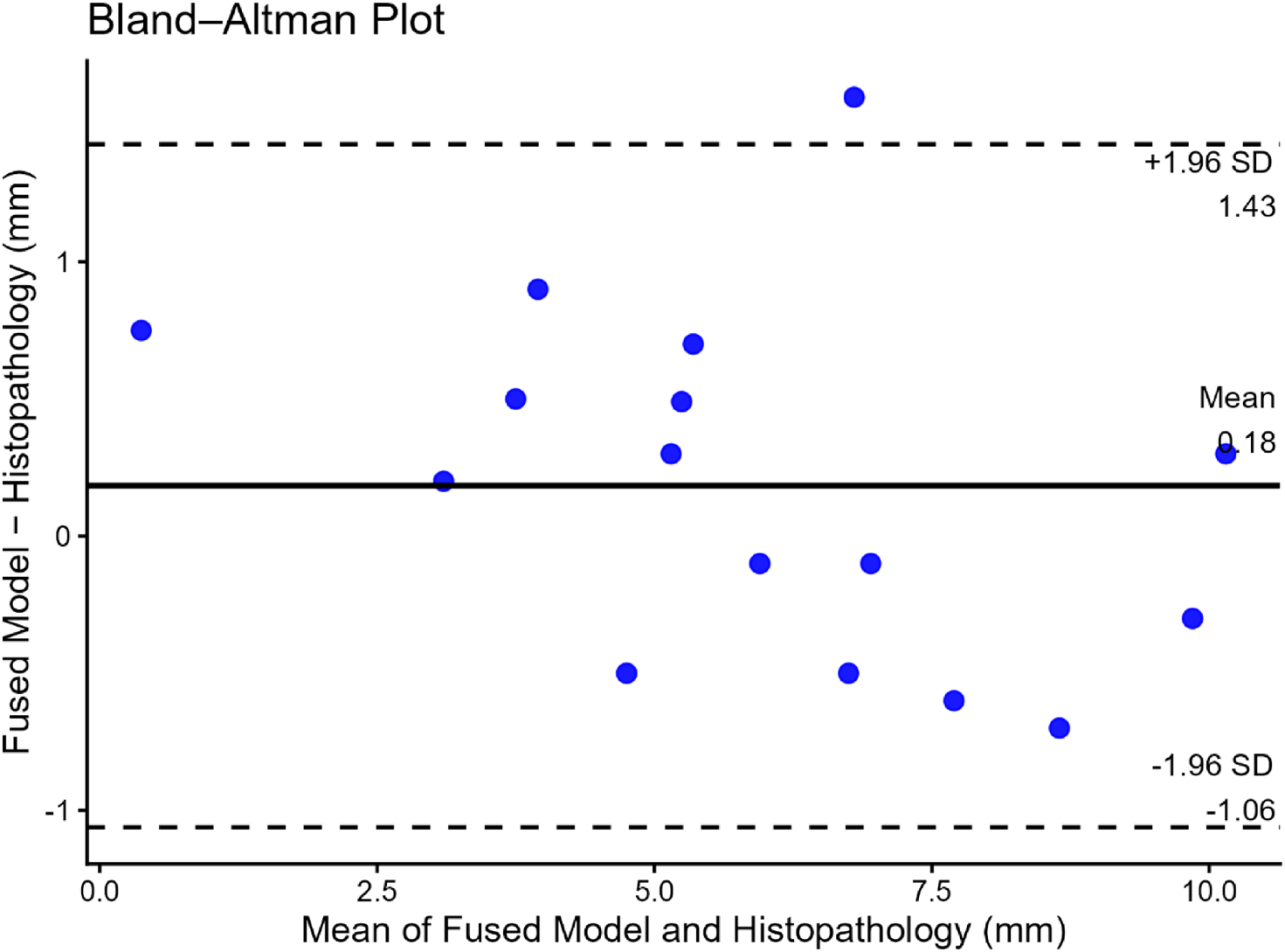
Bland–Altman agreement between fused‐model and histopathology margin distances. Bland–Altman Plot displaying the difference between fused model‐derived and histopathology margin‐distance measurements (Fused Model—Histopathology, mm) plotted against mean of the two methods (mm) for all 16 cases. Blue points represent individual paired measurements while the solid black horizontal line denotes the mean bias. Dashed black lines indicate the 95% limits of agreement (mean bias ±1.96 SD of the differences). [Color figure can be viewed at wileyonlinelibrary.com]

## 4 Discussion

In this study, we describe a novel approach for the co‐registration of intraoperative micro‐PET/CT DICOM data and a 3D specimen map detailing pathologic processing to correlate micro‐PET/CT margin measurements with gold standard histopathology. This protocol was performed without added delays to standard of care gross pathologic processing and allowed for the successful generation of 5 fused models of 4 different head and neck specimen types. The level of agreement between fused model‐derived measurements and histopathology demonstrates that, at least in this early investigation, micro‐PET/CT margin measurements correlate strongly with gold standard histopathology.

Despite the importance of surgical margins in head and neck cancer, methods of intraoperative margin assessment remain limited [17]. Supplementary modalities such as tumor‐target fluoroscopy, optical techniques, and touch imprint cytology have shown similar diagnostic performance compared to FSA [17]. However, these modalities are often limited in their tissue penetration [18, 19] or provide only binary assessments [18, 20], weakening their utility in head and neck cancer where margin‐distance measurements are often critical to adjuvant treatment decisions. Most importantly, these other novel intraoperative modalities lack 3D specimen and margin visualization as well as distance measurement. Micro‐PET/CT has emerged as a novel modality across multiple solid malignancies to enhance intraoperative surgical margin evaluation by providing full 3D specimen visualization and distance measuring [13, 21, 22].

To correlate micro‐PET/CT uptake with histopathology, Debacker et al. utilized frozen sectioning and a custom Python alignment algorithm to co‐register images of micro‐PET/CT uptake to histology slides in head and neck cancer [23]. This algorithm allowed the authors to scale, contour, segment, and rigidly transform images of micro‐PET/CT uptake with histopathology. Maris et al. applied a similar method in breast cancer while training artificial intelligence models to segment tumors and estimate margin distances [24]. In their study, the trained models demonstrated higher sensitivity and specificity for positive margins than simple thresholding models, but evaluation was limited to binary assessment of positive margins rather than margin distance. We extend this work by assessing agreement between continuous margin‐distance measurements derived from fused models and gold standard histopathology. Utilization of annotated models in our study served to ensure measurement was performed from the same margin sites sampled during histopathology. GMM and Kruskal‐Wallis analysis then served the dual purpose of characterizing tumor‐specific uptake and assessing feasibility of accurate margin‐distance measurement. The excellent agreement demonstrates that model‐derived measurements to micro‐PET/CT uptake can be made within ±1.25 mm.

The results of this technique must be contextualized within the limitations of ^18^F‐FDG micro‐PET/CT and the physical properties of the ^18^F tracer. Studies examining theoretically achievable spatial resolution of PET/CT in soft tissue have demonstrated a 2D 90th percentile range of 0.7 mm deviation [25]. Emitted positrons are measured within 0.7 mm of their initial position following ^18^F decay 90% of the time. Thus, margin distances measured within micro‐PET/CT and our fused models have an inherent uncertainty of ±0.7 mm. Nevertheless, while the deviation of ±1.25 mm of this technique is marginally higher than this inherent uncertainty, there is excellent absolute agreement (ICC = 0.97). Both the 95% LoA and small mean bias reported in this manuscript are promising. Furthermore, these findings are consistent with the known FWHM of 1.87 mm of the micro‐PET/CT device. While this pertains to the full scan, it is largely defined by the lower PET resolution while specimen boundaries are defined by the micro‐CT with a resolution of 0.1 mm offering higher precision. As measurements are made to a single point within the PET uptake to the specimen boundary, this localization precision depends largely on the signal‐to‐noise ratio and should not be greatly impacted by the FWHM. The mean SUVmax (18.3, SD 15.4) of our cohort indicates robust uptake and therefore, strong signal allowing for higher precision in measurements. However, this may explain the outlier value which occurred in Case #2 with the lowest SUVmax (3.6), which demonstrated the lowest signal to noise ratio. In routine clinical practice, histopathologic margin‐distance measurements are typically not reported on a sub‐millimetric scale decreasing the practical impact of this limitation. The clinical implications of this level of deviation remain to be seen and its impact is certain to be heightened surrounding critical margin thresholds (1 mm and 5 mm) and thus requires clinician judgment be applied. However, the comprehensive 3D metabolic assessment provided by the micro‐PET/CT scanner offers a promising adjunct to standard of care FSA. Enhanced visualization within 1.25 mm of ground truth may inform the decision to send intraoperative FSA and sites of margin sampling. While further work is required, this presents a promising step in the accuracy and validation of this novel modality.

The retrospective nature of the measurements performed in this study presents a limitation which should be considered when interpreting these results. All measurements were made following pathologist consultation and gold standard measurements were known at the time of model‐derived measurements. To minimize this impact, researchers were limited to a single measurement and analysis for each margin. The unblinded nature of the study may introduce potential observer and confirmation bias conflating the level of agreement and highlights the need for a prospective, blinded study of this technique. The data presented in this manuscript represents only feasibility for accurate model‐derived measurements. Separately, false positives due to inflammation or infection represent a well‐documented limitation of ^18^F‐FDG PET/CT imaging [26]. We attempted to account for this by performing both GMM and Kruskal‐Wallis analysis of complete margin sampling sites to incorporate potential false positive uptake within eventual margin‐distance measurement [14, 27]. Thus, the ±1.25 mm deviation reported can be considered in future studies prospectively utilizing fused models or when seeking to translate these findings to intraoperative measurement [27]. While we utilized an 80% of SUVmax upper‐bound threshold across all measurements based on previous literature [15, 16], this has yet to be validated within head and neck malignancies and is based on a reference standard in the breast cancer literature. PET uptake is further impacted by dose of tracer, timing of infusion and extirpation, and tissue properties which contribute to potential variability. Additionally, thresholding of micro‐PET/CT may greatly impact margin‐distance measurements necessitating standardized thresholding as performed in this study. Given the small sample size of this cohort, the described technique remains untested in the setting of shallow, broad tumors which may occur with superficial mucosal margins. However, the uptake from ^18^F‐FDG has been demonstrated to remain accurate when examined within a frozen section strengthening its possible utility in thin lesions [24]. Furthermore, deep margins represent the key challenge in head and neck cancer with one study noting that deep margins were present in 87% of involved margins [28] while less than 2% of resected specimens had a solely involved mucosal margin [29]. Finally, mucosal margin shrinkage due to formalin fixation may impact the accuracy of this technique as studies have demonstrated a 3.7%–25.6% (0.3 mm–1 mm) reduction in margin distances following fixation [30, 31, 32]. This may present an inherent limitation to this method of comparison, however this emphasizes the importance of characterizing tumor specific uptake and potential utility of intraoperative, pre‐fixation margin assessment. Future studies using artificial intelligence to determine optimal thresholds of micro‐PET/CT may further increase usability. Separately, the upper and lower‐bound thresholds should be further investigated to determine optimal thresholding of micro‐PET/CT in future studies.

This study highlights a novel technique for correlating micro‐PET/CT uptake to histopathology margin sampling sites. If prospectively validated, this technique may improve surgeon understanding of micro‐PET/CT and the accuracy of intraoperative measurement. Micro‐PET/CT may additionally offer a modality for informing sites of margin sampling for pathologists to decrease sampling error on the main specimen. Providing surgeons intraoperative margin distances within ±1.25 mm may inform decisions surrounding FSA which may gain additional benefit from the 3D margin visualization of micro‐PET/CT. Accurate intraoperative measurement may also guide real‐time decisions aimed at balancing functional preservation with adequate oncologic resection by giving surgeons greater confidence in whether additional tissue should be resected.

## 5 Conclusion

This study demonstrates the feasibility of co‐registering micro‐PET/CT DICOM data with 3D specimen models annotated to show sites of margin sampling. Early margin analysis showed strong concordance of micro‐PET/CT margin distance with the gold standard histopathology. Future research will prospectively validate this approach.

## Funding

This work was supported by a National Cancer Institute (NCI) K08 Career Development Award—5K08CA293255‐02.

## Ethics Statement

This study was approved by the Vanderbilt University Medical Center Institutional Review Board IRB#221597 and IRB#242063. The study was conducted in accordance with the ethical standards of the Declaration of Helsinki. Written informed consent was obtained for all patients.

## Supporting information


**Figure S1:** Coronal image on intraoperative micro‐PET/CT and histopathology of Case #2 lateral margin (Left–Right). (Left) Coronal slice of a left mandibulectomy specimen (Case #2) from intraoperative micro‐PET/CT demonstrating the lateral margin (red arrow). (Right) Histopathology of the lateral margin (black arrow) from final pathology report.
**Figure S2:** Axial image on intraoperative micro‐PET/CT and histopathology of Case #3 medial margin (Left–Right). (Left) Axial slice of a right partial glossectomy specimen (Case #3) from intraoperative micro‐PET/CT demonstrating the medial margin (red arrow). (Right) Histopathology of the medial margin (black arrow) from final pathology report.
**Figure S3:** Axial image on intraoperative micro‐PET/CT and histopathology of Case #4 lateral margin (Left–Right). (Left) Axial slice of left maxillectomy specimen (Case #3) from intraoperative micro‐PET/CT demonstrating the lateral margin (red arrow). (Right) Histopathology of the lateral margin (black arrow) from final pathology report.
**Figure S4:** Axial image on intraoperative micro‐PET/CT and histopathology of Case #5 left inferior hypopharynx mucosal margin (Left–Right). (Left) Axial slice of a total laryngectomy specimen (Case #5) from intraoperative micro‐PET/CT demonstrating the left inferior hypopharyngeal margin (red arrow). (Right) Histopathology of the lateral margin (black arrow) from final pathology report.

## Data Availability

Research data are not shared.
