# Supplementary material for: Early Feasibility of Registration of Micro‐PET/CT Scans to Annotated 3D Specimen Models
Source: Head Neck. 2026 Jun 16;48(12):3286–98. doi: 10.1002/hed.70363 (PMC13755738; doi:10.1002/hed.70363)
Supplement: Supplementary file 1 [file HED-48-3286-s001.docx]

**Supplementary Figure 1: Coronal Image on Intraoperative Micro-PET/CT and Histopathology of Case #2 Lateral Margin (Left-Right).** (Left) Coronal slice of a left mandibulectomy specimen (Case #2) from intraoperative micro-PET/CT demonstrating the lateral margin (red arrow). (Right) Histopathology of the lateral margin (black arrow) from final pathology report.

**Supplementary Figure 2: Axial Image on Intraoperative Micro-PET/CT and Histopathology of Case #3 Medial Margin (Left-Right).** (Left) Axial slice of a right partial glossectomy specimen (Case #3) from intraoperative micro-PET/CT demonstrating the medial margin (red arrow). (Right) Histopathology of the medial margin (black arrow) from final pathology report.

**Supplementary Figure 3: Axial Image on Intraoperative Micro-PET/CT and Histopathology of Case #4 Lateral Margin (Left-Right).** (Left) Axial slice of left maxillectomy specimen (Case #3) from intraoperative micro-PET/CT demonstrating the lateral margin (red arrow). (Right) Histopathology of the lateral margin (black arrow) from final pathology report.

**Supplementary Figure 4: Axial Image on Intraoperative Micro-PET/CT and Histopathology of Case #5 Left Inferior Hypopharynx Mucosal Margin (Left-Right).** (Left) Axial slice of a total laryngectomy specimen (Case #5) from intraoperative micro-PET/CT demonstrating the left inferior hypopharyngeal margin (red arrow). (Right) Histopathology of the lateral margin (black arrow) from final pathology report.
